# Positive organizational behavior: Longitudinal effects on subjective well-being

**DOI:** 10.1371/journal.pone.0198588

**Published:** 2018-06-22

**Authors:** Kathrin Heinitz, Timo Lorenz, Daniel Schulze, Julia Schorlemmer

**Affiliations:** 1 Department of Education and Psychology, Freie Universitaet Berlin, Berlin, Germany; 2 Department of Occupational Medicine, Charité Berlin, Berlin, Germany; Universita degli Studi di Bari Aldo Moro, ITALY

## Abstract

Increasing individual subjective well-being has various positive outcomes, knowledge about its antecedents and the mediators of this relationship can therefore help to increase subjective well-being and the accompanying positive effects. The more future oriented facets of psychological capital, i.e. optimism, hope and self-efficacy have been shown in several studies to be positively related to subjective well-being and negatively to ill-being. Furthermore, recent studies suggest coping strategies as mediators for these relationships. In our study, we examined the longitudinal relation of optimism, hope and self-efficacy with subjective well-being and ill-being in a German panel dataset and tested the mediating effect of flexible goal adjustment in a path model. Our results show a statistically significant positive effect of self-efficacy and optimism on subjective well-being as well as a statistically significant negative effect of optimism on depression over three years. All three predictors show a statistically significant relation with flexible goal adjustment, but flexible goal adjustment did not mediate the effect on subjective well-being or depression.

## Introduction

Happy workers will be more productive workers—as Cropanzano and Wright [1] acknowledge, this relation has been supported if happiness was operationalized as well-being, although the happy-productive worker hypothesis [2] has been subjected to much debate and produced controversial empirical results [3]. To learn about mechanisms that increase well-being is relevant in the work context as well as independent of the context [4]. It is relevant for the individual itself, but also, according to the happy productive worker hypothesis, for organizations.

Numerous theories have set out to explain how various individual or job characteristics influence well-being (for an overview see [5]), e.g. the conservation of resources theory [6], the job-demands-resources theory [7] or the stressor-detachment model [8]. According to the conservation of resources theory [6], individuals seek to acquire and maintain resources. Their gain or maintenance results in well-being [9]. Hobfoll [6] distinguishes four types of resources that have the potential to increase well-being: physical objects, conditions, energies or personal characteristics [9]. In our study we focus on personal characteristics and more specifically on characteristics proposed by positive psychology, as well-being can be enhanced by interventions targeting on these constructs [10]. More specifically we examine the longitudinal effects of self-efficacy, optimism and hope on well-being. Coping strategies are being discussed to function as mediating mechanisms of this relation [11]. Flexible goal adjustment as one coping strategy has been shown to mediate the relationship between optimism and well-being in a concurrent sample [11]. We therefore also examine if flexible goal adjustment mediates the relation between the three facets optimism, hope and self-efficacy with well-being over time.

### Positive organizational behavior: Self-efficacy, optimism and hope

Positive psychology in the workplace encompasses various concepts [12] such as positive organizational behavior or positive organizational scholarship. They are united in their focus on strengths and flourishing [13, 14] but have differing perspectives. The focus of positive organizational behavior is on the individual within the organization. Luthans [15] defines micro-level positive organizational behavior as the “study and application of positively oriented human resource strengths and psychological capacities”(p59). Positive organizational behavior in this sense is measurable and meets the open-to-development criterion. It is therefore open to individual learning and change [15–17]. Three of these positive organizational behavior capacities are self-efficacy, optimism, and hope. They all “fit the specific criteria of being positive, unique, measurable, capable of being learned and developed, and manageable for performance improvement”([15] p70). All three are part of the overarching concept of Psychological Capital (PsyCap) [18, 17], including resiliency besides self-efficacy, optimism, and hope. PsyCap was developed as a work-related concept and, as a whole, is defined as “a core psychological factor of positivity in general, and POB criteria meeting states in particular, that go beyond human and social capital to gain a competitive advantage through investment/development of ‘who you are’” ([18] p253). PsyCap influences a variety of outcomes, such as job satisfaction and commitment [19, 20] or reduced absenteeism [21]. Furthermore, individuals high in PsyCap perform better than those low in PsyCap since they can draw upon more resources to pursue goals [22, 23]. Furthermore, PsyCap is linked to an improved psychological and physical well-being [24]. Of the four PsyCap components especially self-efficacy, optimism and hope are goal- and future oriented.

Self-efficacy is a dispositional resource used for coping with all kinds of demands and challenges [25, 26] and can be defined as “one’s beliefs about his or her ability to mobilize the motivation, cognitive resources, and courses of action necessary to execute a specific action within a given context”([27] p66). Optimism as conceptualized within the concept of PsyCap [28] includes not only the dispositional optimistic look towards the future, but also global positive expectations [29, 30]. Following Seligman [14], not only this positive outlook but also the personal reflection of positive and negative events in the past, present and future relevantly influence optimism [30]. Optimistic persons expect positive things to happen and therefore believe in a positive future [31, 32]. Hope is “a positive motivational state that is based on an interactively derived sense of successful agency (goal directed energy) and pathways (planning to meet goals)”([33] p287). Hopeful individuals are able to generate alternative routes in an adaptive way [34, 35]. Goal-directed thoughts lead to positive and active emotions (e.g. curiosity) or negative and passive emotions, depending on past experiences with goal pursuits [36, 35]. Hopeful individuals use the ‘willpower’ to generate personal goals and resolutely follow them and the ‘waypower’ to adapt alternatives while obstacles occur on their way [37]. There has been a lot of research regarding the distinction of optimism and hope [38–42], and while being overlapping and having trait-like thoughts about goals in common [41], their differential relations with outcomes suggest that they are still distinct constructs [39, 43].

### Subjective well-being, ill-being and positive organizational behavior

All three, self-efficacy, hope, and optimism have shown to be related to or predict well-being as well as ill-being. One way of operationalizing well-being is via subjective well-being, which is a multifaceted construct, consisting of affective and cognitive components [44]. It is not only associated with but can also lead to health and longevity [3, 45, 46], increase work-related productivity and success [47], and positively influence social relationships [45, 48]. In contrast to well-being is the term ill-being, which refers to negative psychological constructs such as depression [49]. For the examination of the effects of positive organizational behavior both aspects seem to be relevant, the prevention of ill-being as well as the promotion of subjective well-being, as the absence of ill-being does not automatically lead to subjective well-being and vice versa [50].

Self-efficacy as “the power of believing you can” [51] correlates to and influences well-being positively [25, 52–56], and reduces depression [57–59]. Optimism influences the way people feel and how they actively try to solve problems when being confronted with obstacles and hence influences subjective well-being [60–62]. Practicing optimism leads to increased well-being [63]. Hopeful individuals are motivated to energetically pursue goals and to consider alternative routes to achieve them [37]. They “generate more effective coping strategies to setbacks due to pathways thinking, and thus experience more positive emotions which”([37] p295) and this enhances their well-being. Accordingly, in their meta-analysis and review, Reichard et al. [37] found statistically significant effect sizes between hope and job satisfaction and well-being and negative effect sizes between hope, stress, and burnout.

So far, no study compared the effects of these three central constructs of positive psychology. PsyCap is the only exception, but it is mostly examined as g-factor, effects of its subfacets are seldom shown. PsyCap also has statistically significant relations to well-being [64] and predicts subjective well-being within a three-week period [65]. According to previous research on self-efficacy, optimism and hope with well-being and ill-being, we propose:

(1) Self-efficacy (a), optimism (b) and hope (c) at T_0_ positively predict subjective well-being at T_1_.
(2) Self-efficacy (a), optimism (b) and hope (c) at T_0_ negatively predict depression scores at T_1_.

### Flexible goal-adjustment as a mediator

Goal regulation strategies have been frequently discussed as possible mediators between optimism and well-being [11, 66, 67]. The results of Hanssen et al. [11] suggest that especially flexible goal adjustment applies as a mediator in this relationship. Flexible goal adjustment can be seen as passive accommodative coping, as changing personal preferences due to situational restrictions, which seems to be especially relevant in later life [68]. It is complementary to tenacious goal pursuit, an assimilative coping strategy, in which life circumstances are adjusted to obtain a desired condition [68]. The flexible management of personal goals is associated with well-being [69–73]. Goal regulation strategies such as flexible goal adjustment were found to affect the quality of life and general well-being of patients with chronic illnesses [74, 75]. Furthermore, in older age flexible people report higher levels of self-esteem [76] and are more likely to look at their biographical past in a self-enhancing way [77]. By disengaging old goals when necessary, reengaging new goals and accepting this change by adjusting to the situation, people can stay optimistic and hopeful despite witnessed difficulties and are more satisfied with their lives [68, 71]. At first sight, high self-efficacy should inhibit flexible goal adjustment, in a sense that if one is certain about one’s possibilities, there is no need to change a goal. But as Brandtstädter ([78] p143) argued, it might not be that simple, as “if getting what one wants is central to the concept of power, it follows that a way to retain a sense of efficacy may be to adjust one’s preferences to the range of the feasible”. Following this argumentation, the relation of self-efficacy and subjective well-being as well as depression, should also be mediated by flexible goal adjustment.

We therefore propose:

(3) Flexible goal adjustment mediates the effect of self-efficacy (a), optimism (b) and hope (c) at T_0_ on subjective well-being at T_1_.
(4) Flexible goal adjustment mediates the effect of self-efficacy (a), optimism (b) and hope (c) at T_0_ on depression at T_1_.

## Method

### Participants and procedure

Data for this study comes from the German Ageing Survey [79], an ongoing register-based, cohort-sequential, nationwide representative survey of the German population aged 40 to 85. The present study used the sample of 2008 (6205 participants) and 2011. In 2011, 2858 participants of the original sample were re-interviewed with a response rate of 64.4%. The response rate corresponds to that of other longitudinal studies with comparable age groups [80]. Participants completed face-to-face interviews and a self-administered questionnaire. Due to the longitudinal data analysis only people who were interviewed 2008 and 2011, with employment status working and maximum age 65 (maximum retirement age in Germany) were included. These criteria excluded 1858 persons from the data analysis. Furthermore, 408 were excluded due to missing data (345 due to declined answers on relevant variables, 63 due to high amount (> 50%) of missing data scale wise). The total sample included 592 people between 39–62 years of age in 2008 (M = 49.43, SD = 5.60), and nearly half of them (47%) were men.

### Measures

Self-Efficacy was measured using the Generalized Self-Efficacy Scale [81]. The scale consists of 5 items (e.g. “I can usually handle whatever comes my way”) with a 4-point Likert response format (Cronbach’s α_2008_ = .79; α_2011_ = .79).

Optimism was measured using the ‘Affective valence of future time perspective’ scale [82]. The scale consists of five items (e.g. “I look to the future with confidence”) with a 4-point Likert response format (Cronbach’s α_2008_ = .86; α_2011_ = .86).

Hope was measured using the Hope Scale [83]. The scale consists of 8 items (e.g. “There are lots of ways around any problem”) with a 4-point Likert response format (Cronbach’s α_2008_ = .83; α_2011_ = .83).

Flexible goal adjustment was measured using the Flexible Goal Adjustment Scale [84]. The scale consists of 10 items (e.g. “I adapt quite easily to changes in plans or circumstances”) with a 5-point Likert response format (Cronbach’s α_2008_ = .77; α_2011_ = .77).

Subjective well-being was composed of the three factors positive affect, negative affect, and life satisfaction [44]. The affective component of subjective well-being was measured using the German version [85] of the brief Positive Affect Negative Affect Scale (PANAS) [86]. The scale consists of 10 items, 5 negative adjectives (e.g. “ashamed”, Cronbach’s α_2008_ = .87; α_2011_ = .86) and 5 positive adjectives (e.g. “enthusiastic”, Cronbach’s α_2008_ = .85; α_2011_ = .86) with a 5-point Likert response format.

The cognitive component of subjective well-being was measured using Satisfaction with Life Scale [87]. The scale consists of 5 items (e.g. “I am satisfied with my life”) with a 5-point Likert response format (Cronbach’s α_2008_ = .84; α_2011_ = .85).

Depressive symptoms were measured using the General Depression Scale (Allgemeine Depressions Skala) [88]. The scale consists of 15 items (e.g. “I felt that everything I did was an effort”) with a 4-point Likert response format (Cronbach’s α_2008_ = .85; α_2011_ = .87).

More details on the questionnaires used can be found at www.dza.de/en/research/deas/research-instruments.html.

### Covariates / Control variables

Sex is an important covariate for subjective well-being, as it has been revealed that especially older women reported lower subjective well-being [89] and there are differential relations for sex between our predictors and subjective well-being [37]. Income and education are positively related to subjective well-being [90]. The socio-economic status (SES) in general affects physical and psychological health negatively [91, 92]. SES is assessed by the individual´s occupation and classified with the International Socio-Economic Index of Occupational Status (ISEI), which scales occupations by relating them to education and income [93]. SES influences subjective well-being and health through different ways: Low SES groups show higher health risk behavior [94], have less access to health care and receive qualitatively poorer health care [95], the material deprivation is higher and the psychosocial environment is more stressful over the life course [96]. Further we included the occurrence of special health events such as an accident or a serious illness due to the possible impact of these events on subjective well-being and depression [97].

### Data analysis

We checked the demographic data for effects of systematic drop-outs due to the large gap between complete and final data set for our analysis. Only minimal changes in age, sex and the distribution of individuals in East and West Germany could be found.

As subjective well-being consisted of three independently measured constructs, we tested for the unidimensionality of the compound variable using confirmatory factor analysis. The Satorra-Bentler adjusted χ^2^ was calculated to adjust for non-normal distributions of the variables [98]. The fit was inspected using the criteria proposed by Hu and Bentler [99]. According to these indices the model for subjective well-being in 2008 (Satorra-Bentler-χ^2^ (1, 592) = .025, p = .874, CFI = 1.00, SRMR = .003, RMSEA = .00, CI_RMSEA_ = .00 - .06) as well as the model for SWB in 2011 (Satorra-Bentler-χ^2^ (1, 592) = .052, p = .820, CFI = 1.00, SRMR < .001, RMSEA < .001, CI_RMSEA_ = .00 - .06) showed a good model fit. To test our hypotheses we used a structural equation model (SEM). The data analysis was run using the statistical software R [100]. The confirmatory factor analyses and SEM were conducted using the “lavaan” package [101].

## Results

**Fig 1:**
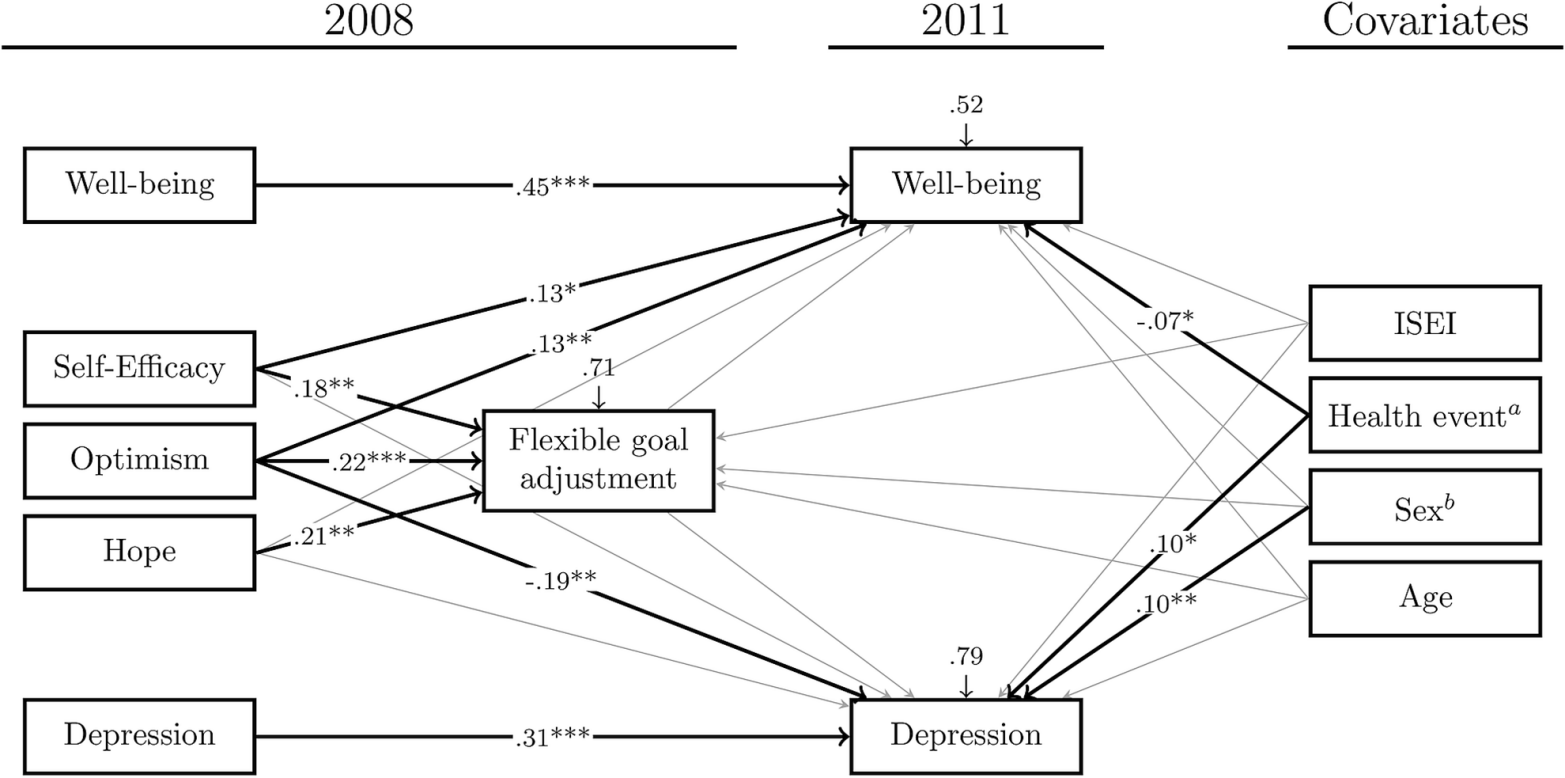
Path model of the longitudinal relation of self-efficacy, optimism and hope with well-being and depression, mediated by flexible goal-adjustment. Regression weights are standardized beta-weights, ^a^ critical health event is coded no/yes; ^b^ sex is coded male/female;.* p < .05, ** p < .01, *** p < .001.

Table 1 offers a general overview over the bivariate correlations of all variables used in this study. The path model to test our hypotheses shows an acceptable model fit, Satorra-Bentler-χ^2^ (5, 592) = 17.952, p = .003, CFI = .979, SRMR = .015, RMSEA = .066, CI_RMSEA_ = .037 - .098. Fig 1 shows the tested model with highlighted statistically significant paths while all direct regression paths of the model are displayed in Table 2. Self-efficacy and optimism at T_0_ both statistically significantly predict subjective well-being at T_1_, but only optimism at T_0_ shows statistically significant negative relations to depression at T_1_. Hope at T_0_ shows no statistically significant relation to either subjective well-being or depression at T_1_. Hypotheses 1(c) and 2(c) therefore have to be rejected.

**Table 1:** Summary of the bivariate correlations of all variables.

	1	2	3	4	5	6	7	8	9	10	11
1) Age	1										
2) Sex	-.08	1									
3) ISEI	.05	.00	1								
4) Flex 08	.00	.01	.04	1							
5) Hope 08	-.01	-.02	.17***	.49***	1						
6) Optimism 08	-.03	.00	.19***	.46***	.64***	1					
7) Self-efficacy 08	-.02	-.02	.13**	.47***	.80***	.59***	1				
8) SWB 08	.01	.05	.19***	.38***	.66***	.63***	.58***	1			
9) Depression 08	.01	.09*	-.08	-.18***	-.24***	-.30***	-.23***	-.38***	1		
10) SWB 11	.06	-.03	.16***	.36***	.54***	.54***	.52***	.66***	-.37***	1	
11) Depression 11	.02	.12**	-.11**	-.14***	-.20***	-.29***	-.22***	-.30***	.42***	-.46***	1
12) Health Event	.01	-.03	-.02	-.03	-.05	-.07	-.04	-.10*	.09	-.13***	.14***

*Note*. ISEI = socio-economic index of occupational status, Flex 08 = flexible goal-adjustment 2008, SWB 08 = subjective well-being 2008, SWB 11 = subjective well-being 2011; sex is coded male/female; critical health event is coded no/yes

* p < .05

** p < .01

*** p < .001.

**Table 2:** Regression paths using MLM estimator.

Parameter estimate	unstandardized	standard error	standardized	p-score
Flexible goal adjustment 08				
Hope 08	.262	.083	.211	.002
Optimism 08	.206	.046	.229	< .001
Self-efficacy 08	.219	.074	.179	.003
Sex	.016	.034	.017	.628
Age	.001	.003	.017	.629
ISEI	-.002	.001	-.060	.078
Depression score 11				
Depression score 08	.329	.063	.305	< .001
Flexible goal adjustment 08	.009	.043	.011	.834
Hope 08	.082	.067	.081	.219
Optimism 08	-.140	.046	-.190	.003
Self-efficacy 08	-.094	.066	-.095	.153
Sex	.075	.029	.095	.009
Age	.001	.003	.016	.649
ISEI	-.001	.001	-.057	.152
Critical health event	.111	.054	.098	.040
Subjective well-being 11				
Subjective well-being 08	.423	.045	.447	< .001
Flexible goal adjustment 08	.102	.075	.059	.173
Hope 08	.049	.138	.023	.725
Optimism 08	.202	.075	.130	.007
Self-efficacy 08	.282	.114	.133	.014
Sex	-.091	.050	-.054	.072
Age	.008	.005	.055	.079
ISEI	.002	.002	.027	.387
Critical health event	-.173	.073	-.072	.018

Notes: ISEI = socio-economic index of occupational status, χ_SB_^2^ (5, 592) = 17.952, p = .003, CFI = .979, SRMR = .015, RMSEA = .066, CI_RMSEA_ = .037 - .098; sex is coded male/female; critical health event is coded no/yes.

Whereas all three predictors are statistically significantly related to flexible goal adjustment at T_0_, no indirect path is statistically significant with standardized regression paths between .002-.01. These results are not in favor of hypotheses 3 and 4.

## Discussion

Positive organizational behavior focuses on individual strengths and resources [15], optimism, hope and self-efficacy being prominent examples. All three are related to positive aspects from an individual perspective but also from an organizational perspective. We therefore wanted to examine if those three aspects have a longitudinal relation with subjective well-being and ill-being and if this relation is mediated by the coping strategy flexible goal adjustment. For our study we used a working subsample of the German Ageing Survey [79].

In accordance with other studies, our results show that all three facets are correlated to subjective well-being and depression concurrently [38, 102, 103]. According to the results of our path model, hope seems not to be relevant for time-lagged subjective well-being nor depression, but optimism predicts both and self-efficacy predicts subjective well-being after a three year period. Hence, in a direct comparison, optimism seems to be the most relevant for subjective well-being and depression of the three constructs.

One reason for the diminishing effect of hope could be the chosen outcome criterion. Bryant and Cvengros [39] argue that optimism focuses more on broader quality of future outcomes whereas hope focuses more on specific goals. As well-being in our study with the two components of affect and general life satisfaction presents rather a general positive than a goal-specific outcome, our results are in accordance with their argument. This applies accordingly for our second outcome variable depression. On the other hand, our results also raise the question if the impact of hope diminishes because optimism and self-efficacy have too much overlap with the waypower and willpower components of hope [33]. In the study of Bryant and Cvengros [39] for example, as well as in other studies [103] the three variables are highly correlated. Bryant and Cvengros [39] argue that hope and optimism have discriminant relations with other variables, but can also be considered as having a common g-factor. The meta-analytical results in the study of Alarcon et al. [38] accordingly point out, that optimism and hope are distinguishable but related and that they have discriminant relations with outcome variables, with optimism being more relevant in situations with low personal control and hope being more relevant in situations with higher personal control. Further research with different outcome variables is therefore needed to clarify if hope in comparison to optimism has more impact on time-lagged rather goal specific outcomes.

Our results are also in line with Benyamini and Roziner [104] who found a statistically significant relation between optimism and self-rated health on life satisfaction after a five year period. In their study this relation was eliminated when affectivity was included in the analysis. In our study, however, affect is a part of the outcome as the broader concept of subjective well-being includes life satisfaction as well as positive and negative affect. Optimism might therefore be more responsible for the variance in the affective part of our subjective well-being measure.

Self-efficacy was also related to subjective well-being over a three year period in our sample. This is in support of previous studies that see self-efficacy as an important factor for well-being [53]. However, we found no relation with ill-being. Although depression goes in hand with lower levels of self-efficacy [105], higher levels of self-efficacy seem not to impact levels of depression significantly. This result contradicts the findings of Holahan and Holahan [58] and Maciejewski et al. [59]. It should be stated though, that the study of Holahan and Holahan [58] focuses on self-efficacy for social support. Social support on the other hand also has an impact on depression [58, 106]. Maciejewski et al. [59] examined general self-efficacy. Their effect size for the indirect effect of baseline self-efficacy on follow-up depression for the subsample without prior depression (β_indirect_ = -0.114, s.e. = 0.033, *p* < 0.001) resembles ours (β_indirect_ = -0.095, s.e. = 0.066, *p* = 0.153), but Maciejewski et al. [59] have a much larger sample. Furthermore, overlaps between hope, optimism and self-efficacy have been controlled for in our sample, which might reduce the effect of self-efficacy. Taken together, the results suggest that there is a possible small effect of self-efficacy on later depression.

Concurrently, optimism, hope and self-efficacy were all correlated with flexible goal adjustment. Contrary to the results of Hanssen et al. [11] however, flexible goal adjustment did not mediate the relation between optimism and self-efficacy with well-being nor with depression over a three-year period. Hence, flexible goal adjustment might have cross-sectional assocations with well-being [11] but no long-time mediating effect.

The substantially long period of time (three years in our study) might be a drawback as adjusted goals might rather be accepted as new goals. In order to examine the mediating effects of flexible goal adjustment in the relation of positive organizational behavior and subjective well-being respectively ill-being, longitudinal designs with shorter time periods could therefore be appropriate. Furthermore, tenacious goal pursuit [66] might be an alternative for the long-time mediation, as the modification of behavior or the situation in order to fit with one’s goals might rather be the appropriate long-term strategy resulting in higher levels of subjective well-being and lower levels of ill-being. Following the argumentation of Heyl, Wahl, and Mollenkopf [72] both flexible goal adjustment and tenacious goal pursuit are necessary for an adaptive self-regulation and both have differential effects on the affective aspects of subjective well-being. In order to get a complete picture, both aspects as well as their interactions should be considered.

### Practical implications

Subjective well-being has many desirable effects on a personal but also on the organizational level (e.g. health and longevity, [45], or work-related productivity, [47]). Knowing about antecedents of subjective well-being, and especially about developable factors is therefore helpful in many ways. Positive organizational behavior subsumes developable factors that have an effect on organizational well-being, optimism, hope and self-efficacy being among them. There are intervention programs, even online interventions, in order to develop these facets, e.g. the interventions to strengthen psychological capital [107, 20], that can support subjective well-being and reduce ill-being. Focussing especially on optimism and self-efficacy can help to develop easy to handle and little time-consuming interventions and strengthen the organizations following a people-based approch as e.g. described by Manuti and de Palma [108].

Furthermore, subjective well-being has an impact on the retention intentions of older workers [109]. Supporting an optimistic outlook and self-efficacy in older workers might therefore be one part of the puzzle to retain them in the active workforce in order to face the demographic change. One has to bear in mind, however, that optimism and self-efficacy do not inherently have a positive effect, but that this effect depends on the context [110]. Therefore, “the best interventions to promote well-being may thus be those that teach people different skills […] and the best time and place to use each one” ([111] p577).

### Limitations

As we used the panel data of the German Ageing Survey [79], we were restricted in our measures as well as in the age range of our participants. We had to rely on the measures used in the German Ageing Survey and they proved to have satisfying internal consistency scores, but we have to point out, that our results only hold for the measures used. Other measures that focus on different aspects might yield different results [38]. Furthermore, our results are specific for an older population. As we mentioned, certain coping strategies might be more relevant in later life [78, 68], implications for younger populations therefore cannot be deviated and need further research.

Also we used context-free measures of well-being. Referring to the happy-productive worker hypothesis, and the remarks made by Cropanzano and Wright [1], these overall measures of well-being have an impact on work outcomes. This relation is empirically supported e.g. by Tsai, Chen, and Liu [112] or Fritz and Sonnentag [113] (see also [114]). One has to note, however, that the effects are smaller than those between context-specific measures and work performance. All in all, context-specific and context-free measures of well-being are overlapping to a large extent [44], which is especially the case for the working context as work takes a large amount of time. Investing in possibilities to enhance general well-being should pay off for organizations. Furthermore, the work context can be a place full of opportunities in order to enhance self-efficacy.

Another possible limitation of this study is the issue of self-report measures and common method variance [115]. This issue is heavily debated in work and organizational psychology with unclear results if common method is in fact a problem to the results in this field or how big the problem might be [116–119]. Even with the open debate we think that it is worth mentioning that it could be a possible limitation.
